# Relationships between cognitive performance, clinical insight and regional brain volumes in schizophrenia

**DOI:** 10.1038/s41537-022-00243-x

**Published:** 2022-04-04

**Authors:** Erkan Alkan, Simon L. H. Evans

**Affiliations:** Faculty of Health and Medical Sciences, University of Surrey, Guildford, Surrey, UK

**Keywords:** Schizophrenia, Human behaviour

## Abstract

Impairments in cognitive performance are common in schizophrenia, and these contribute to poor awareness of symptoms and treatment (‘clinical insight’), which is an important predictor of functional outcome. Although relationships between cognitive impairment and reductions in regional brain volumes in patients are relatively well characterised, less is known about the brain structural correlates of clinical insight. To address this gap, we aimed to explore brain structural correlates of cognitive performance and clinical insight in the same sample. 108 patients with schizophrenia (SZH) and 94 age and gender-matched controls (CON) (from the Northwestern University Schizophrenia Data and Software Tool (NUSDAST) database) were included. SZH had smaller grey matter volume across most fronto-temporal regions and significantly poorer performance on all cognitive domains. Multiple regression showed that higher positive symptoms and poorer attention were significant predictors of insight in SZH; however, no significant correlations were seen between clinical insight and regional brain volumes. In contrast, symptomology did not contribute to cognitive performance, but robust positive relationships were found between regional grey matter volumes in fronto-temporal regions and cognitive performance (particularly executive function). Many of these appeared to be unique to SZH as they were not observed in CON. Findings suggest that while there exists a tight link between cognitive functioning and neuropathological processes affecting gross brain anatomy in SZH, this is not the case for clinical insight. Instead, clinical insight levels seem to be influenced by symptomology, attentional performance and other subject-specific variables.

## Introduction

Persistent impairments in cognitive performance are detectable in most patients with schizophrenia (SZH) and have important consequences for functional capacity^1^. Cognitive deficits span most neurocognitive domains, including attention^2,3^, working memory^4,5^, episodic memory^6,7^ and executive function^8–10^. Cognitive deficits have also been observed in first-episode patients, suggesting they are not due to drug treatment^11,12^; indeed, cognitive impairments are largely unimproved by pharmacological therapy^13^. Further, cognitive impairments have been observed in unaffected relatives of SZH; meta-analyses indicate cognitive deficits in unaffected first-degree relatives versus controls across a variety of cognitive tasks and particularly those tapping executive function^14^. In terms of underlying neurobiological mechanisms, brain structural correlates of impaired cognitive performance in SZH have been identified. Reduced grey matter volume in fronto-temporal regions has been consistently implicated^15,16^. For example, Guo et al.^17^ identified a correlation between poorer executive function and smaller left inferior frontal volume in treatment-naïve, first-episode SZH, which seemed to be unique to patients as it was not evident in controls. Likewise, Minatogawa-Chang et al.^18^ also identified correlations between overall cognitive performance and volumes in dorsolateral and inferior frontal gyri, and left superior temporal cortex, that were unique to patients, while Yasuda et al.^19^ found that cognitively impaired SZH had lower prefrontal, left superior temporal and left insula volumes, compared to a relatively cognitively preserved SZH group and controls.

In addition to neurocognitive impairments, schizophrenia is also characterised by a limited capacity for ‘clinical insight’^20–22^, which refers to a patient’s awareness of symptoms, their need for treatment, and the effects of medication^20,23^. Clinical insight is an important but understudied variable in schizophrenia, it correlates with functional capacity in patients^24^ and is predictive of longer-term outcomes^25^. Also, cognitive deficits have been shown to contribute to impaired insight is in SZH, with executive dysfunction particularly implicated^26–28^. For example, poor insight correlates with perseverative errors made on the Wisconsin Card Sorting Test (WCST)^29,30^. Performance in other cognitive domains has also been linked to insight in SZH, including social cognition^31^ and attention^32,33^, as well as overall neurocognitive performance^31,34,35^. As one might expect, symptom severity has also been consistently shown to impact insight^20,22,36,37^ as cognitive and perceptual distortions (i.e. delusions and hallucinations) undermine insight capacity^37^.

Despite its importance for determining functional outcome in SZH, clinical insight is relatively understudied in SZH. Cognition-brain structure relationships in SZH have been well investigated, but only a handful of studies have attempted to identify relationships between brain structural indices and clinical insight in SZH. Smaller grey matter volume in dorsolateral prefrontal cortex (DLPFC) has been linked to poor insight in first-episode patients^38,39^. In chronic SZH, Flashman et al.^40^ focused on frontal subregions in a sample of 15 SZH and reported correlations between insight and volume in middle frontal gyrus, while Cooke et al.^41^ found correlations between the ability to recognise experiences as abnormal and volume in the total and right superior temporal gyrus in 52 SZH. Cooke et al.^41^ also reported a strong relationship between a patient’s awareness of problems and volume of left praecuneus, and a relationship between symptom re-labelling and total brain volume. Likewise, Sapara et al.^42^ classified 40 SZH into poor (*N* = 20) and preserved insight (*N* = 20) groups: smaller fronto-temporal and parahippocampal volumes characterised the poor insight group. However, other studies have found no significant correlations: Bassitt et al.^43^ reported no significant relationships between insight and prefrontal volumes in 50 SZH, while Béland et al.^44^ found no correlation between clinical insight, subcortical volumes and cortical thickness measures in a sample of 110 SZH. Thus, as noted by a recent meta-analysis, evidence for structure-insight correlations is inconsistent: even when effects were detected, effect sizes tended to be low to moderate in size, this could be due to other contributing factors (e.g. social aspects)^45^, and since many studies used limited sample sizes, further work is needed.

Despite inconsistencies in findings, however, there is some evidence to suggest that brain volumes within fronto-temporal regions might represent the brain structural correlates of clinical insight. Volume-insight relationships thus potentially overlap with those of neurocognitive performance. However, neuroimaging studies have examined these constructs individually, making it difficult to draw conclusions regarding the degree of overlap. To our knowledge, no study has analysed relationships with brain volumes for both clinical insight and neurocognitive measures within the same sample. High levels of heterogeneity between SZH study samples and methods make direct comparisons between studies difficult. Investigating the structural correlates of both cognitive performance and insight within a single, well-powered sample, to allow inferences regarding the differential contributions of regional brain volumes to each of these constructs in SZH, is therefore of value. In this study, we examine links between regional grey matter volume, clinical insight, and a comprehensive set of neurocognitive performance measures in a sample of 108 SZH. Hypothesising that symptom severity and cognitive impairment would explain a significant amount of variance in clinical insight, we first ran regression models to test this. Then we performed correlation analyses to identify grey matter volume correlates of clinical insight and cognitive performance, hypothesising that fronto-temporal grey matter volumes would show relationships with both cognitive performance and clinical insight in SZH. To contextualise the findings and characterise the cohort under study, we also compared cognitive performance and regional grey matter volumes against an age- and gender-matched control group.

## Results

### Sample characteristics

Demographics are reported in Table 1. For education, SZH (*M* = 11.93, SD = 2.22) had a significantly lower level compared to CON (*M* = 14.34, SD = 2.67, *p* < 0.01). For age, there were no significant differences between SZH (*M* = 33.7, SD = 12.7) and CON (*M* = 30.61, SD = 13.26), and there were no differences in gender balance (all *p* values > 0.05). We report descriptive statistics for SUMD (by subdomain, and total) in Table 1. Considerable variance in scores was present in the sample (e.g. on SUMD total, *M* = 14.23, S.D. = 7.37)

**Table 1:** Demographic characteristics of the sample.

	CON (*N* = 94)	SZH (*N* = 108)	*p*-value
Age, *M* (SD)	30.61 (13.26)	33.70 (12.70)	0.092^a^
Female (*N*, %)	41 (43.6%)	34 (31.5%)	0.075^b^
Education (years of schooling) (SD)	14.34 (2.67)	11.93 (2.22)	<0.001^a^
Medication (chlorpromazine equivalent mg/day) (SD)	N/A	434.33 (399.75)	N/A
SAPS (mean) (SD)	N/A	16.72 (13.63)	N/A
SANS (mean) (SD)	N/A	22.48 (15.07)	N/A
Clinical Insight (SUMD Total) (mean) (SD)	N/A	14.23 (7.37)	N/A
Awareness of Mental Disorder (Past)	N/A	2.87 (1.18)	N/A
Awareness of Mental Disorder (Current)	N/A	1.95 (1.23)	N/A
Awareness of Medication Effectiveness (Past)	N/A	2.56 (1.90)	N/A
Awareness of Medication Effectiveness (Current)	N/A	1.92 (1.36)	N/A
Awareness of Social Consequences (Past)	N/A	2.78 (1.97)	N/A
Awareness of Social Consequences (Current)	N/A	2.15 (1.42)	N/A

*CON* healthy controls, *SZH* schizophrenia, *M (SD)* mean (standard deviation), *SAPS* Scale for the Assessment of Positive Symptoms, *SANS* Scale for the Assessment of Negative Symptoms, *SUMD* The Scale to Assess Unawareness in Mental Disorder, *N/A* not applicable.

^a^Independent sample *t* test.

^b^Pearson chi-square.

### SZH vs CON comparisons

#### Cognitive Domain Scores

**Fig. 1:**
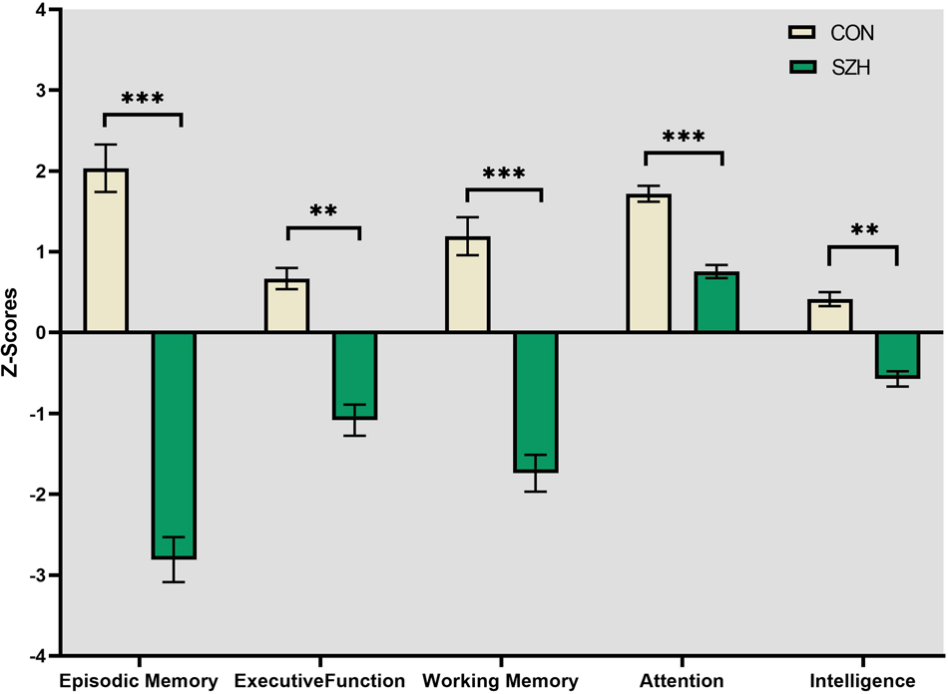
Group Differences in Cognitive Scores between Controls (CON) and Schizophrenia Patients (SZH). ***p < 001, **p < 0.01.

ANCOVAs (age, gender and education as covariates) showed that SZH had significantly lower performance on all cognitive domain scores (Fig. 1, *p* < 0.01, FDR corrected).

#### ICV-adjusted Volumes

**Fig. 2:**
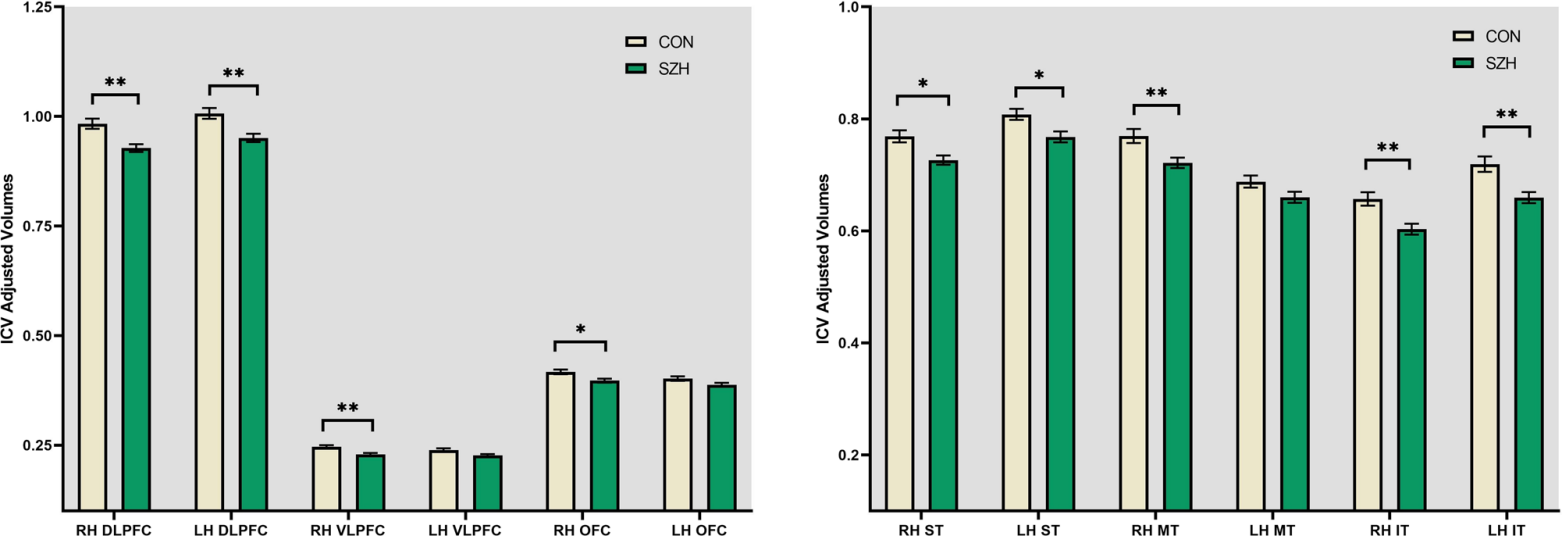
Group differences in ICV-Adjusted Volumes between Controls (CON) and Schizophrenia Patients (SZH). RH right hemisphere, LH left hemisphere, DLPFC dorsolateral prefrontal cortex, VLPFC ventrolateral prefrontal cortex, OFC orbitofrontal cortex, ST superior temporal, MT middle temporal, IT inferior temporal. **p < 0. *01*, *p < 0.*05*.

In frontal ROIs, ANCOVA (age and gender as covariates) showed that SZH had reduced ICV-adjusted volumes in bilateral DLPFC (right: unadjusted *p* = 0.001, left: unadjusted *p* = 0.003), right VLPFC (unadjusted *p* = 0.003) and right OFC (unadjusted *p* = 0.044) compared to CON (Fig. 2). After FDR correction for multiple comparisons, differences in bilateral DLPFC and right VLPFC remained significant, but those in right OFC did not.

In temporal ROIs, SZH had reduced volumes in bilateral superior temporal (right: unadjusted *p* = 0.026, left: unadjusted *p* = 0.045) right middle temporal (unadjusted *p* = 0.009), and bilateral inferior temporal (right: unadjusted *p* = 0.003, left: unadjusted *p* = 0.003) compared to CON. After FDR correction, differences in right superior temporal, bilateral inferior temporal, and right middle temporal remained significant (all p values < 0.05, FDR corrected), while left superior temporal survived at trend (*p* = 0.054, FDR corrected) (Fig. 2).

### Multiple regression analyses in SZH

#### Clinical insight (SUMD scores)

To investigate factors influencing clinical insight (SUMD total scores and SUMD current subscales) in SZH, multiple regression analysis was performed using the enter method. Gender, duration of illness, years of schooling, medication (chlorpromazine equivalent), SAPS, SANS, cognitive domain scores and crystalized intelligence were entered as predictors. The overall model was statistically significant and explained 21.4% of the total variance (*F* = 2.191, *p* = 0.037, *R*^2^ = 0.214). Higher positive symptoms from SAPS (β = 0.320, *p* = 0.025), and lower attention scores (*β* = −0.731, *p* < 0.001) were the significant variables, predicting poorer insight (Supplementary Table 1). Regarding subscales of SUMD, duration of illness was the only predictor of current awareness of mental disorder (*p* = 0.046), but the overall model was not statistically significant (*p* = 0.058) (Supplementary Table 1). There was no statistically significant predictor of current awareness of medication effectiveness or current awareness of social consequences of the disorder (all *p* values > 0.05).

### Correlations with ROI brain volumes in SZH

#### Cognitive performance

Partial correlations (controlling for duration of illness, medication and education) were performed to investigate relationships between neurocognitive performance and ICV-adjusted volume within the ROIs in SZH. Partial correlations (controlling for age and education) were also performed to investigate relationships between neurocognitive performance and ICV-adjusted volume within the ROIs in CON.

##### Executive function

**Fig. 3:**
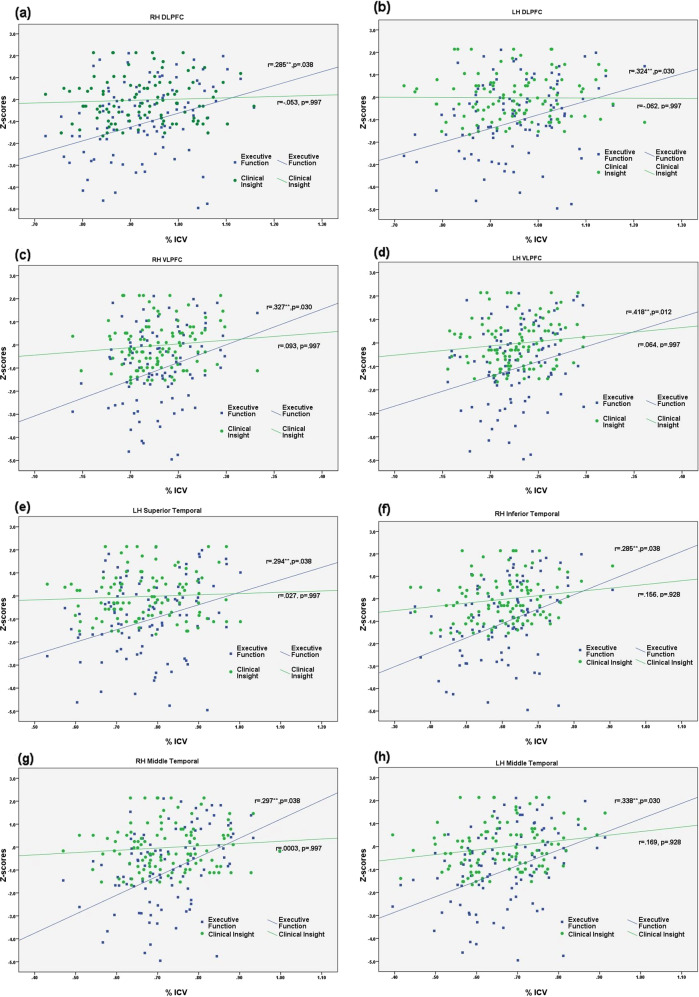
Correlations between ICV-adjusted volumes and cognitive performance and clinical insight. Scatter plots demonstrating significant positive relationships between cognitive domains and ICV-adjusted volumes, adjusted for duration of illness, medication and education in SZH. Executive function scores positively correlated with volume in bilateral DLPFC (**a**. right: *p* = 0.038, left: **b**. *p* = 0.030), bilateral VLPFC (**c**. right: *p* = 0.030 and **d**. left: *p* = 0.012), left superior temporal (**e**. *p* = 0.038), right inferior temporal (**f**. *p* = 0.038), and bilateral middle temporal (right: **g**. *p* = 0.038 and left: **h**. *p* = 0.030). **FDR corrected *p* < 0.05.

Executive function scores positively correlated with volume in bilateral DLPFC (right: *p* = 0.038, left: *p* = 0.030), bilateral VLPFC (right: *p* = 0.030, left: *p* = 0.012), left superior temporal (*p* = 0.038), right inferior temporal (*p* = 0.038), and bilateral middle temporal (right: *p* = 0.038 and left: *p* = 0.030), and these relationships survived FDR correction in SZH (Table 2, Fig. 3). No associations were seen in CON (Supplementary Table 2). A percentile bootstrap method was used to test differences in correlations between SZH and CON groups. A significant difference in correlations was found for all the aforementioned regions apart from left VLPFC (Supplementary Table 3). A percentile bootstrap method was also used to test differences in correlations between executive function and clinical insight on ICV-Adjusted volumes. A significant difference in correlations was found for all the aforementioned regions (Supplementary Table 4).

**Table 2:** Correlations Between Cognitive Scores and ICV-adjusted volumes in SZH (controlling for duration of illness, education and medication).

ICV-adjusted volumes	Executive function			Attention			Episodic memory			Working memory		
	*r*	*P*_unadjusted_	*P*_FDR_	*r*	*P*_unadjusted_	*P*_FDR_	*r*	*P*_unadjusted_	*P*_*FDR*_	*r*	*P*_unadjusted_	*P*_FDR_
Right dorsolateral prefrontal	0.285^a^	0.025	0.038	0.183	0.213	0.256	−0.016	0.900	0.942	−0.037	0.775	0.775
Left dorsolateral prefrontal	0.324^a^	0.010	0.030	0.199	0.176	0.256	−0.031	0.807	0.942	0.052	0.689	0.775
Right ventrolateral prefrontal	0.327^a^	0.009	0.030	0.352	0.014	0.154	0.095	0.458	0.942	0.277	0.029	0.264
Left ventrolateral prefrontal	0.418^a^	0.001	0.012	0.264	0.069	0.154	0.119	0.354	0.942	0.115	0.374	0.690
Right orbitofrontal	0.140	0.279	0.372	0.155	0.294	0.321	0.037	0.771	0.942	0.095	0.460	0.690
Left orbitofrontal	0.063	0.626	0.626	0.183	0.213	0.256	0.056	0.661	0.942	−0.045	0.728	0.775
Right superior temporal	0.097	0.451	0.492	0.267	0.066	0.154	0.185	0.146	0.942	−0.157	0.222	0.595
Left superior temporal	0.294^a^	0.020	0.038	0.283	0.051	0.154	0.102	0.426	0.942	−0.081	0.529	0.705
Right inferior temporal	0.285^a^	0.025	0.038	0.208	0.156	0.256	0.035	0.785	0.942	0.149	0.248	0.595
Left inferior temporal	0.129	0.319	0.383	0.115	0.435	0.435	−0.009	0.942	0.942	0.108	0.404	0.690
Right middle temporal	0.297^a^	0.019	0.038	0.321	0.026	0.154	−0.054	0.676	0.942	0.186	0.147	0.588
Left middle temporal	0.338^a^	0.007	0.030	0.257	0.077	0.154	0.044	0.729	0.942	0.256	0.044	0.264

*SZH* schizophrenia.

^a^FDR corrected *p* < 0.05.

##### Episodic Memory

No associations were seen between episodic memory and volume within any ROI either in SZH (Table 2) or CON (Supplementary Table 2).

##### Working Memory

Scores in SZH positively correlated with right VLPFC volume (uncorrected *p* = 0.029) and left middle temporal volume (uncorrected *p* = 0.044), although these did not survive for multiple comparisons (Table 2). In CON, working memory scores negatively correlated with right VLPFC volume (uncorrected *p* = 0.034), however, this did not survive FDR correction (Supplementary Table 2). Between-groups, a significant difference in correlations was found for volume in right VLPFC and left middle temporal (Supplementary Table 3).

##### Attention

Scores in SZH positively correlated with volumes in right VLPFC (uncorrected *p* = 0.014) and right middle temporal (uncorrected *p* = 0.026), however, these did not survive FDR correction (Table 2). In controls, there was a negative correlation between attention scores and volumes in right middle temporal (uncorrected *p* = 0.046), however, this did not survive FDR correction (Supplementary Table 2). Between-groups, a significant difference in correlations was found for volume in right VLPFC and right middle temporal (Supplementary Table 4).

### Clinical insight

No significant correlations were seen between clinical insight (SUMD) and any ICV-adjusted ROI volumes (Table 3). As a follow-up confirmatory analysis, we also compared the regional ICV-adjusted grey matter volumes between two subgroups of SZH, defined by taking the lowest and highest quartiles based on total SUMD scores. Results revealed no significant differences between these two subgroups in any ROIs. Further, we looked at correlations with each of the three SUMD subscale scores individually (awareness of (i) having a mental disorder, (ii) effects of medication and (iii) social consequences)) and again no correlations with brain volumes were observed (Supplementary Tables 5–6). To check for any relationships between clinical insight and brain volumes outside of the ROIs, an exploratory whole-brain approach was tried assessing correlations across all regional volumes output by Freesurfer, no statistically significant relationships were identified (Supplementary Table 7).

**Table 3:** Correlations between ICV-adjusted volumes and SUMD in SZH (controlling for duration of illness, education and medication).

ICV-adjusted volumes	SUMD		
	*r*	*P*_unadjusted_	*P*_FDR_
Right dorsolateral prefrontal	−0.053	0.679	0.997
Left dorsolateral prefrontal	−0.062	0.626	0.997
Right ventrolateral prefrontal	0.093	0.464	0.997
Left ventrolateral prefrontal	0.064	0.616	0.997
Right orbitofrontal	−0.009	0.942	0.997
Left orbitofrontal	−0.020	0.877	0.997
Right superior temporal	−0.152	0.232	0.928
Left superior temporal	0.027	0.833	0.997
Right inferior temporal	0.156	0.218	0.928
Left inferior temporal	0.037	0.770	0.997
Right middle temporal	0.000	0.997	0.997
Left middle temporal	0.169	0.181	0.928

*SZH* schizophrenia, *SUMD* The Scale to Assess Unawareness in Mental Disorder.

## Discussion

This study set out to better characterise the factors influencing, and the interrelationships between, cognitive performance and clinical insight in SZH. Regression models examined predictor variables, correlations with brain volumes within fronto-temporal regions were then assessed. Also, cognitive performance and brain volumes in SZH were contrasted against an age- and gender-matched control group to contextualise the findings: as expected, SZH had poorer cognitive performance across all domains and lower grey matter volume within most of the ROIs. Impaired neurocognitive performance^2–10^ and reduced grey matter volume in fronto-temporal regions^46–50^ are reliably reported in the literature.

Regarding clinical insight, multiple regression analyses linked higher attention scores (indexed using a sustained attention task) and lower positive symptom severity to better insight in SZH, explaining over 20% of the variance. Sustained attention has been previously linked to unawareness of illness in SZH^32,33^. Although not found here, other studies have linked better executive function (measured by WCST perseverative errors)^30^ and visual working memory (measured by a visual tracking test called the Situational Awareness Test)^51^ to higher insight. Lysaker et al.^52^ suggest that attention impairments, alongside deficits in memory and executive function, undermine a patient’s abilities to retrieve, integrate and reflect on relevant information: insight requires being able to recognise and make links between experiences salient to the illness, and poorer attention could undermine this. Regarding symptom severity, effects on clinical insight in SZH is well-demonstrated^22,36,37^, high levels of delusions or hallucinations symptomatology may limit patients’ capacity to understand their symptoms and diagnosis due to the disruptive, disturbing nature of these complex, incomprehensible and potentially traumatic unusual mental events^37,52^. Nevertheless, in a metanalysis, Mintz et al.^22^ found that this relationship might be only minimal; thus, other related factors including premorbid functioning should be considered when investigating clinical insight.

Previous studies point to the possibility of overlapping neural substrates underlying cognitive performance and clinical insight: volumes within fronto-temporal brain regions have been implicated by prior work, but no prior study has investigated relationships within the same sample. Results revealed various significant relationships between fronto-temporal brain volumes and cognition in SZH, particularly for executive function performance, which is associated with larger grey matter volume in bilateral DLPFC, bilateral VLPFC, bilateral middle temporal, left superior temporal and right inferior temporal regions, in line with previous studies^53–55^. These were only observed in the SZH group, not controls: the difference in correlations between groups was significant. This highlights the importance of these regional volumes for explaining variation in patients’ cognitive ability, particularly in executive function: while the initial between-group analyses showed that these regions were smaller in the SZH group as a whole, individual differences in volumes impact cognitive performance levels in patients only. Likewise, Guo et al.^17^ found relationships between inferior frontal volume and executive function in SZH that were not present in controls. Brain structural abnormalities in SZH have been linked to executive function, particularly for DLPFC^8^; superior temporal gyrus (STG) has also been implicated^56^. Although not typically regarded as being central for executive functioning, it has been suggested that in SZH, abnormalities in STG could undermine the ability to identify and classify task-relevant information^15^. Temporal volumes seem to be of high importance for cognition performance in SZH, evidence of relationships unique to SZH have been found. Hartberg et al.^57^ found that cortical thickness in middle and transverse temporal correlated with processing speed in SZH (but not controls), while Ehrlich et al.^58^ found that working memory correlated with thickness in right middle and superior temporal gyrus in SZH, rather than lateral prefrontal cortex as seen in controls. Here, we found left middle temporal volume to correlate with working memory and sustained attention performance in SZH only, and while these did not survive multiple comparisons correction, the correlations were significantly different from those present in controls. Decreased or reversed anatomical asymmetry in the temporal lobe^59^ could be a contributing factor^60^. Also, greater involvement of temporal regions in SZH could reflect the engagement of additional regions to compensate for prefrontal cortical dysfunction^61^. Consistent with this, Minatogawa-Chang et al.^18^ found relationships between overall cognitive performance and volume in superior temporal regions, as well as DLPFC and inferior frontal, that were unique to SZH and not present in controls. Similar results have been shown by recent work using cortical thickness measures^62^.

Taken together, findings highlight the importance of preserved fronto-temporal brain volumes for cognitive performance in SZH; the current study and others point to specific relationships that are unique to SZH, suggesting that disease-related neuropathological processes affecting gross brain anatomy directly undermine cognitive functioning in SZH. Moreover, the most robust relationships identified were between executive function and frontal and temporal regional volumes in SZH. The stability^63^ and trait-like nature of executive dysfunction may explain why the most robust relationships were observed between executive impairments and grey matter reductions in fronto-temporal regions compared to other cognitive domains. However, it should be noted that these results should ideally be replicated in a control group that is more comparable to SZH in terms of variance and ability in cognitive performance, to determine whether these cognition-structure relationships are in fact specific to SZH and not just due to higher cognitive impairment and heterogeneity in patients. Also, it should be noted that the ROI approach used in this study meant that only a limited (albeit well-justified) set of brain regions were considered in the analyses: this meant that relationships outside of the search area might have been missed and should be considered a limitation of the current work.

In contrast, no correlations with clinical insight were observed for any of the ROIs. Our findings differ from some previous studies showing structure-clinical insight relationships^38–42,64^. However, better-powered studies using sample sizes similar to ours have mostly found no correlation between grey matter volume and clinical insight^43,44,65,66^, in line with our findings. In their meta-analysis, Pijnenborg et al.^45^ highlights the risk of false positives based on small to modest sample sizes: in the studies showing insight-structure relationships, SZH sample sizes ranged from 14 to 52^38–42,64^ while studies showing no correlations have samples ranging from 50 to 141^43,44,65,66^. Inconsistent findings regarding neural correlates of insight might also be due to methodological differences such as different instruments used to measure insight, sample characteristics and inconsistent definitions of insight^52^. In some of the studies that reported structure-insight relationship, insight was measured either by a single item (i.e. Shad et al.^38^ who used a single item, derived from the Hamilton Depression Rating Scale) or a self-rated questionnaire (e.g. the Birchwood Insight Scale was used by Sapara et al.^42^). We used a well-validated clinician-rated scale (SUMD) to assess insight, many other studies using clinician-rated scales also found no correlation between insight and brain structure^43,65,66^, although some have^41^. Self-report insight scales have been criticised regarding their accuracy^67^, as they lack the objectivity of a clinician-rated appraisal^68^. Also, other important factors likely contribute to insight, which might overshadow any underlying structural relationships. McFarland et al.^65^ point to the cumulative effects of medication exposure over the course of illness which may obscure possible correlations: they found structure-insight correlations in an FEP sample but not in chronic SZH. Overall, it seems that relationships are more apparent in FEP^38,39,69^ perhaps because pronounced volumetric changes to fronto-temporal regions occur early in the disease course^70^, while over time and as the disease progresses, insight is subsequently influenced by other factors that then obscures these underlying links to brain structure. Here, the regression analyses pointed to the role of attentional capacity and positive symptom severity in poorer insight; thus, fluctuations in symptomology could be important. Also, the level of functional capacity might contribute. Insight in SZH seems to have a bidirectional relationship with functioning. Impaired clinical insight correlates with lower functional capacity in middle-aged and older SZH^24^, and has been linked to poorer social and occupational functioning in FEP^71^. Moreover, a longitudinal study in SZH found that improvements in insight following antipsychotic drug therapy were associated with decreased symptomology and improvement in global functioning^72^. Various studies show that insight correlates with better long-term functioning, but this might be due to its association with symptoms, better medication adherence, and better relationship with the treating clinicians^73,74^. Furthermore, some authors suggest that individual differences in insight might reflect differences in coping strategies. Mintz et al.^22^ argued that lack of insight or denial of the illness could function as a defence mechanism; the denial model suggests that patients who avoid insight through denial might suffer lower levels of distress and maintain better self-esteem^75^. Clearly, the insight concept is complex and multifactorial, and the factors underlying it are yet to be fully elucidated. To this end, Lysaker et al.^52^ proposes an integrative model which specifies that insight involves complex and multiple interactions between various factors, including internal and external circumstances, life trajectory and social factors such as the perceptions of others.

In conclusion, the results of the current study provide insights into the relationships between clinical insight, cognitive performance and regional brain volumes in a relatively large sample of SZH. Widespread cognitive impairments and reduced grey matter volume across fronto-temporal regions were observed in SZH. Sustained attention and positive symptom severity explained variance in insight, but symptomology didn’t influence cognitive performance. Instead, brain volumes in fronto-temporal regions showed strong relationships with cognitive measures that were unique to SZH, highlighting the importance of these for determining cognitive performance levels in patients. In contrast, no volumetric relationships with clinical insight were identified, suggesting that symptomology and other factors are more important, particularly as the disease progresses.

## Methods

### Participants

The current study included 108 SZH (34 females, aged between 17 and 61, mean age 33.7), and 94 age- and gender-matched controls (CON) (41 females, aged between 14 and 66, mean age 30.61), see Table 1. The data were obtained from the publicly available NUSDAST database^76^ and downloaded from the http://schizconnect.org website. The research centre defined exclusion criteria as having an intellectual disability based on DSM-IV, having a severe medical disorder or head injury, and having met the criteria for substance use based on DSM- IV, and written informed consent was obtained from all participants before participation^77^. More information on data sampling and recruitment has been described elsewhere^76^. All SZH were stabilised on antipsychotics for at least two weeks prior to the study^77,78^, medication dose was converted to chlorpromazine equivalents (see Table 1).

### MRI acquisition

NUSDAST collected MRI scans with a Siemens 1.5T Vision Scanner. The details of the acquisition process are described elsewhere^76,78^. Following parameters were defined by the research centre to collect high-resolution T1-weighted structural images using an MPRAGE sequence: TR = 9.7 ms, TE = 4 ms, flip = 10◦, ACQ = 1, 256 × 256 matrix, 1 × 1 mm in-plane resolution, 128 slices, slice thickness 1.25 mm, 5:36 min scan time each^78^. All images were processed via FreeSurfer 3.0.4^79^ and the cortical parcellations were derived based on the Destrieux atlas^80^. Regional grey matter volumes were derived from the Destrieux parcellation. We calculated intra-cranial (ICV)-adjusted volumes by dividing the volume in each ROI by the total ICV and multiplying by 100 ((volume in ROI/ICV) *100).

### Measures

To assess symptom severity, The Scale for the Assessment of Positive Symptoms (SAPS)^81^ and the Scale for the Assessment of Negative Symptoms (SANS)^82^ were used. To assess clinical insight, we used the clinician-rated Scale to Assess Unawareness of Mental Disorder (SUMD)^20^. Following a semi-structured interview, the clinician scores the patient on three items that assess past and current awareness of: (i) having a mental disorder, (ii) effects of medication and (iii) consequences of the mental disorder; items are rated on a five-point Likert-type scale (1 = full awareness, 5 = unawareness). The SUMD total score is the total sum of all past and current scores across these three items.

All participants completed a comprehensive neuropsychological battery across four cognitive domains previously shown to be impaired in SZH, namely, attention^83^, executive function^8^, episodic memory^6^ and working memory^4^. Tasks were as follows.

Continuous Performance Test- Identical Pairs (CPT-IP). A computerised test of sustained attention requires the participant to identify repetition amongst a sequence of nonsense shapes and digit numbers^84,85^. Participants make a button press when two successive stimuli are identical. As a standardised score of accuracy, d-prime is the ratio between hits and false alarms^86^.

Wechsler Memory Scale—Third Edition (WMS-III). Digit Span (total forwards and backwards): participants recite back a sequence of numbers in the same order, and in reverse order^87^. Spatial Span (total forwards and backwards): participants are asked to repeat a spatial sequence demonstrated by examiner in the same order (forward) and reverse order (backward)^88^. Letter Number Sequencing: participants are asked to repeat a mixed list of letters and numbers in alphabetic and ascending orders^88^. Logical Memory subtest: participants are asked to verbally recall a given story immediately (LM I, immediate version) and after a delay interval (LM II, delayed version)^88^, and Family Pictures subtest: subjects are shown a series of pictures of scenarios which participants are required to recall^88^.

Wisconsin Card Sorting Test (WCST)^89^: subjects sort 64 cards based on the colour, shape and numbers, perseverative errors occur when a participant persists in using wrong rule despite negative feedback^90^.

Wechsler Adult Intelligence Scale^91^. Matrix Reasoning subtest: participants select images for pattern completion^92^. Vocabulary subtest: subjects define presented words^93^.

All scores (apart from d-prime from the CPT-IP) were standardised and converted to Z-scores; we then created summary scores for the following cognitive domains:

- Attention: CPT-IP (d prime)
- Working Memory: Sum of *z*-scores from Wechsler Memory Scale—Third Edition (WMS-III); including Digit Span (total forward and backwards), Spatial Span (total forward and backwards) and Letter-Number Sequencing
- Episodic Memory: Sum of *z*-scores from WMS-III Logical Memory and Family Pictures subtests
- Executive Function: Sum of *z*-scores from perseverative errors on the WCST, and WAIS-III Matrix Reasoning subtest

We also used scores from the Vocabulary subtest of the Wechsler Adult Intelligence Scale to assess Crystallized Intelligence as a measure of premorbid IQ^94^.

### ROI approach

The ROIs focused on frontal and temporal regions as previous work has implicated these as the volumetric correlates of cognitive performance (and potentially clinical insight) in SZH. Frontal regions included were: DLPFC (as shown by Jirsaraie et al.^53^, and by Rüsch et al.^54^, measure: executive function; and Minatogawa-Chang et al.^18^, measure: overall cognitive performance), Ventrolateral Prefrontal Cortex (VLPFC) (as shown by Guo et al.^17^, measure: executive function; Minatogawa-Chang et al.^18^, measure: overall cognitive performance, and Goghari et al.^95^, measure: working memory), and Orbitofrontal Cortex (OFC) (as shown by Matsui et al.^96^, measure: semantic memory). Temporal regions included were: superior temporal (as shown by Minatogawa-Chang et al.^18^ and Yasuda et al.^19^, measure: overall cognitive performance), inferior temporal (as shown by Wolf et al.^97^, measure: working memory), and middle temporal (as shown by Wolf et al.^97^, measure: attention). We defined the frontal ROI volumes by combining areas as follows: DLPFC: superior frontal, rostral and caudal middle frontal, VLPFC: pars opercularis, pars orbitalis and pars triangularis, OFC: lateral and medial orbital frontal. To supplement the ROI approach and capture any potentially important relationships outside the pre-defined search area, we additionally ran correlation analyses across all brain regional volumes output by Freesurfer based on the Destrieux atlas, these are reported in supplementary materials.

### Statistical analysis

All cognitive, clinical, and MRI data were analysed using IBM SPSS Statistics 21.0. On demographic variables, groups were compared using independent sample t-tests (for age and education), and a *χ*2 test (for gender). On cognitive performance, groups were compared using Analyses of covariance (ANCOVAs) with age, gender, and years of schooling as covariates. Between-group comparisons in ICV-adjusted volumes were performed using ANCOVA in which age and gender were included as covariates. To correct for multiple comparisons, false discovery rate (FDR) with the Benjamini-Hochberg method was applied^98^ by using FDR online calculator which is freely available on https://www.sdmproject.com/utilities/?show=FDR.

To assess factors that explain variance in clinical insight in the SZH group, a series of multiple regression models were constructed with SUMD subscale current scores and SUMD Total scores as dependent variables (separate models). Gender, duration of illness, years of schooling, medication (chlorpromazine equivalent), SAPS, SANS, cognitive domain scores and crystalized intelligence were entered as independent variables to predict scores on SUMD.

In SZH, partial correlations were performed to assess relationships between ICV-adjusted volumes and cognitive performance and clinical insight, controlling for duration of illness, years of education, and medication. In CON, partial correlations were performed to assess relationships between ICV-adjusted volumes and cognitive performance, controlling for age and years of education. To control for multiple comparisons, FDR corrections were applied as above. The significance threshold was set at *p* < 0.05 (2 tailed) for all analyses.

A percentile bootstrap method^99^ with 5000 iterations was used to test differences in correlations between SZH and CON groups. This was applied when a significant correlation was found in one of the groups (FDR-corrected or unadjusted). A significant difference in correlations is inferred if the 95% percentile bootstrap confidence intervals do not overlap with zero. This analysis was performed in Matlab using code adapted from https://github.com/GRousselet/blog/tree/master/comp2dcorr. This approach is recommended as it is more robust than other methods^100^.

## Supplementary information

SUPPLEMENTAL MATERIAL

## Data Availability

The data that support the findings of this study are available from the corresponding author upon reasonable request.
