# Supplementary material for: Relationships between cognitive performance, clinical insight and regional brain volumes in schizophrenia
Source: Schizophrenia (Heidelb). 2022 Apr 4;8(1):33. doi: 10.1038/s41537-022-00243-x (PMC9261092; doi:10.1038/s41537-022-00243-x)
Supplement: Supplementary file 1 — SUPPLEMENTAL MATERIAL [file 41537_2022_243_MOESM1_ESM.docx]

**Relationships between Cognitive Performance, Clinical Insight and Regional Brain Volumes in Schizophrenia**

**Erkan Alkan****^1^, Simon Evans*^1^**

^1^ Faculty of Health and Medical Sciences, University of Surrey, Guildford, Surrey, United Kingdom

*To whom correspondence should be addressed; Faculty of Health and Medical Sciences, University of Surrey, Guildford, Surrey GU2 7XH, United Kingdom: Tel: +44 (0)1483 686945, email: se0016@surrey.ac.uk

| **Supplementary Table 1.**  Results of Multiple Linear Regression Analysis for Variables Predicting SUMD and Subscale Scores in SZH | | | | | | | | |
| --- | --- | --- | --- | --- | --- | --- | --- | --- |
|  | SUMD Total | | Mental Disorder | | Medication Effectiveness | | Social Consequences | |
| ***Variable*** | ***β*** | ***p*** | ***β*** | ***p*** | ***β*** | ***p*** | ***β*** | ***p*** |
| Gender | .037 | .804 | -.128 | .404 | .045 | .794 | -.049 | .777 |
| Duration of Illness | -.103 | .468 | -.296* | .046 | -.077 | .631 | -.176 | .287 |
| Years of Schooling | -.108 | .565 | -.128 | .502 | -.096 | .650 | -.152 | .482 |
| Medication | -.025 | .857 | .072 | .612 | -.232 | .149 | -.160 | .324 |
| SANS | .132 | .399 | .320 | .050 | .271 | .130 | .186 | .304 |
| SAPS | .320* | .025 | .075 | .593 | .089 | .570 | .102 | .524 |
| Attention | -.731** | <.001 | -.312 | .157 | -.444 | .072 | -.260 | .295 |
| Executive Function | .230 | .165 | .332 | .052 | .297 | .115 | .253 | .185 |
| Episodic Memory | .055 | .734 | -.022 | .892 | -.035 | .848 | -.064 | .733 |
| Working Memory | .440 | .078 | -.114 | .645 | .259 | .347 | -.077 | .783 |
| Crystalized Intelligence | -.107 | .631 | .127 | .577 | .023 | .929 | .236 | .361 |
| **p< .05. **p<.01*  *Abbreviations*. SZH, Schizophrenia; SAPS, Scale for the Assessment of Positive Symptoms; SANS, Scale for the Assessment of Negative Symptoms; SUMD, The Scale to Assess Unawareness in Mental Disorder | | | | | | | | |

| **Supplementary Table 2.**  Correlations Between Cognitive Performance Scores and ICV Adjusted Volumes in CON (controlling for age and education) | | | | | | | | | | | | | |
| --- | --- | --- | --- | --- | --- | --- | --- | --- | --- | --- | --- | --- | --- |
| ICV Adjusted Volumes | **Executive Function** | | | **Attention** | | | **Episodic Memory** | | | **Working Memory** | | | |
|  | *r* | *P_unadjusted_* | *P_FDR_* | *r* | *P_unadjusted_* | *P_FDR_* | *r* | *P_unadjusted_* | *P_FDR_* | *r* | *P_unadjusted_* | *P_FDR_* |  |
| Right Dorsolateral Prefrontal | -0.046 | 0.669 | 0.952 | -0.101 | 0.365 | 0.626 | 0.133 | 0.209 | 0.668 | -0.076 | 0.500 | 0.545 |  |
| Left Dorsolateral Prefrontal | -0.110 | 0.303 | 0.952 | -0.158 | 0.156 | 0.522 | 0.113 | 0.288 | 0.668 | -0.145 | 0.193 | 0.384 |  |
| Right Ventrolateral Prefrontal | -0.073 | 0.494 | 0.952 | -0.066 | 0.556 | 0.741 | 0.006 | 0.954 | 0.954 | -0.234 | 0.034 | 0.384 |  |
| Left Ventrolateral Prefrontal | 0.047 | 0.661 | 0.952 | 0.031 | 0.786 | 0.857 | 0.052 | 0.622 | 0.829 | -0.032 | 0.772 | 0.772 |  |
| Right Orbitofrontal | -0.021 | 0.844 | 0.952 | -0.114 | 0.306 | 0.612 | -0.032 | 0.763 | 0.916 | -0.147 | 0.187 | 0.384 |  |
| Left Orbitofrontal | 0.009 | 0.935 | 0.952 | -0.137 | 0.221 | 0.530 | -0.011 | 0.914 | 0.954 | -0.115 | 0.302 | 0.425 |  |
| Right Superior Temporal | -0.028 | 0.797 | 0.952 | -0.076 | 0.497 | 0.741 | 0.130 | 0.219 | 0.668 | -0.136 | 0.223 | 0.384 |  |
| Left Superior Temporal | 0.045 | 0.678 | 0.952 | -0.033 | 0.771 | 0.857 | 0.080 | 0.452 | 0.678 | -0.111 | 0.319 | 0.425 |  |
| Right Inferior Temporal | -0.041 | 0.703 | 0.952 | -0.019 | 0.867 | 0.867 | 0.119 | 0.260 | 0.668 | -0.077 | 0.493 | 0.545 |  |
| Left Inferior Temporal | -0.126 | 0.240 | 0.952 | -0.152 | 0.174 | 0.522 | 0.102 | 0.334 | 0.668 | -0.147 | 0.188 | 0.384 |  |
| Right Middle Temporal | -0.006 | 0.952 | 0.952 | -0.255 | 0.046 | 0.252 | 0.125 | 0.238 | 0.668 | -0.136 | 0.224 | 0.384 |  |
| Left Middle Temporal | -0.124 | 0.246 | 0.952 | -0.219 | 0.066 | 0.288 | 0.090 | 0.395 | 0.677 | -0.194 | 0.080 | 0.384 |  |
| **FDR corrected p<0.05 | | | | | | | | | | | | | |

| **Supplementary Table 3.**  Group Differences in Correlations between Cognitive Performance and ICV Adjusted Volumes | | |
| --- | --- | --- |
|  | **Bootstrapping (95% CI)** | |
| **Executive Function** | **Lower** | **Upper** |
| Right Dorsolateral Prefrontal | 0.23 | 0.35 |
| Left Dorsolateral Prefrontal | 0.21 | 0.33 |
| Right Ventrolateral Prefrontal | 0.11 | 0.22 |
| Left Superior Temporal | 0.06 | 0.18 |
| Right inferior Temporal | 0.32 | 0.43 |
| Right Middle Temporal | 0.27 | 0.38 |
| Left Middle Temporal | 0.33 | 0.44 |
| **Working Memory** |  |  |
| Right Ventrolateral Prefrontal | 0.24 | 0.36 |
| Right Middle Temporal | 0.14 | 0.26 |
| **Attention** |  |  |
| Right Ventrolateral Prefrontal | 0.19 | 0.31 |
| Left Middle Temporal | 0.42 | 0.53 |
| *Abbreviations,* CI, confidence interval | | |

| **Supplementary Table 4.**  Differences in Correlations between Executive Function and Clinical Insight on ICV Adjusted Volumes | | |
| --- | --- | --- |
|  | **Bootstrapping (95% CI)** | |
|  | **Lower** | **Upper** |
| Right Dorsolateral Prefrontal | 0.22 | 0.34 |
| Left Dorsolateral Prefrontal | 0.25 | 0.37 |
| Right Ventrolateral Prefrontal | 0.07 | 0.19 |
| Left Superior Temporal | 0.14 | 0.26 |
| Right Inferior Temporal | 0.22 | 0.33 |
| Right Middle Temporal | 0.29 | 0.40 |
| Left Middle Temporal | 0.16 | 0.27 |
| *Abbreviations,* CI, confidence interval | | |

| **Supplementary Table 5.**  Correlations Between SUMD Subscale Scores (Current) and ICV Adjusted Volumes in SZH (controlling for duration of illness, education, and medication) | | | | | | | | | | |
| --- | --- | --- | --- | --- | --- | --- | --- | --- | --- | --- |
| ICV Adjusted Volumes | Mental Disorder | | | Medication Effectiveness | | | Social Consequences | | |  |
|  | *r* | *P_unadjusted_* | *P_FDR_* | *r* | *P_unadjusted_* | *P_FDR_* | *r* | *P_unadjusted_* | *P_FDR_* |  |
| Right Dorsolateral Prefrontal | 0.061 | 0.633 | 0.955 | 0.031 | 0.810 | 0.982 | 0.017 | 0.896 | 0.955 |  |
| Left Dorsolateral Prefrontal | 0.016 | 0.898 | 0.955 | -0.062 | 0.625 | 0.982 | -0.020 | 0.873 | 0.955 |  |
| Right Ventrolateral Prefrontal | 0.027 | 0.831 | 0.955 | -0.007 | 0.958 | 0.982 | 0.075 | 0.556 | 0.955 |  |
| Left Ventrolateral Prefrontal | 0.041 | 0.748 | 0.955 | -0.063 | 0.621 | 0.982 | 0.126 | 0.321 | 0.955 |  |
| Right Orbitofrontal | -0.039 | 0.760 | 0.955 | -0.018 | 0.886 | 0.982 | -0.099 | 0.437 | 0.955 |  |
| Left Orbitofrontal | -0.007 | 0.955 | 0.955 | 0.003 | 0.982 | 0.982 | -0.089 | 0.486 | 0.955 |  |
| Right Superior Temporal | 0.108 | 0.397 | 0.955 | -0.015 | 0.908 | 0.982 | -0.024 | 0.852 | 0.955 |  |
| Left Superior Temporal | 0.076 | 0.552 | 0.955 | 0.058 | 0.649 | 0.982 | -0.007 | 0.955 | 0.955 |  |
| Right Inferior Temporal | 0.215 | 0.088 | 0.955 | 0.161 | 0.203 | 0.982 | 0.166 | 0.189 | 0.955 |  |
| Left Inferior Temporal | 0.149 | 0.241 | 0.955 | 0.113 | 0.372 | 0.982 | 0.010 | 0.940 | 0.955 |  |
| Right Middle Temporal | 0.061 | 0.631 | 0.955 | 0.051 | 0.688 | 0.982 | 0.044 | 0.728 | 0.955 |  |
| Left Middle Temporal | 0.102 | 0.424 | 0.955 | 0.054 | 0.669 | 0.982 | 0.170 | 0.179 | 0.955 |  |
| Abbrevations: SUMD, The Scale to Assess Unawareness in Mental Disorder; SZH, Patients with Schizophrenia | | | | | | | | | |  |

| **Supplementary Table 6.**  Correlations Between SUMD Subscale Scores (Past) and ICV Adjusted Volumes in SZH (controlling for duration of illness, education, and medication) | | | | | | | | | | |
| --- | --- | --- | --- | --- | --- | --- | --- | --- | --- | --- |
| ICV Adjusted Volumes | Mental Disorder | | | Medication Effectiveness | | | Social Consequences | | |  |
|  | *r* | *P_unadjusted_* | *P_FDR_* | *r* | *P_unadjusted_* | *P_FDR_* | *r* | *P_unadjusted_* | *P_FDR_* |  |
| Right Dorsolateral Prefrontal | -0.044 | 0.731 | 0.986 | -0.115 | 0.364 | 0.728 | -0.051 | 0.692 | 0.765 |  |
| Left Dorsolateral Prefrontal | -0.085 | 0.502 | 0.731 | -0.189 | 0.136 | 0.728 | -0.116 | 0.362 | 0.765 |  |
| Right Ventrolateral Prefrontal | 0.016 | 0.899 | 0.502 | -0.061 | 0.634 | 0.844 | 0.072 | 0.574 | 0.765 |  |
| Left Ventrolateral Prefrontal | 0.038 | 0.767 | 0.899 | -0.049 | 0.703 | 0.844 | 0.110 | 0.386 | 0.765 |  |
| Right Orbitofrontal | 0.033 | 0.799 | 0.767 | -0.143 | 0.260 | 0.728 | -0.060 | 0.640 | 0.765 |  |
| Left Orbitofrontal | -0.035 | 0.785 | 0.799 | -0.168 | 0.185 | 0.728 | -0.072 | 0.571 | 0.765 |  |
| Right Superior Temporal | -0.021 | 0.868 | 0.785 | -0.176 | 0.165 | 0.728 | -0.111 | 0.381 | 0.765 |  |
| Left Superior Temporal | 0.012 | 0.924 | 0.868 | -0.121 | 0.342 | 0.728 | -0.083 | 0.513 | 0.765 |  |
| Right Inferior Temporal | 0.058 | 0.649 | 0.924 | 0.012 | 0.924 | 0.924 | 0.023 | 0.859 | 0.765 |  |
| Left Inferior Temporal | -0.035 | 0.785 | 0.649 | -0.049 | 0.700 | 0.844 | -0.115 | 0.364 | 0.765 |  |
| Right Middle Temporal | -0.002 | 0.986 | 0.785 | 0.017 | 0.897 | 0.924 | -0.049 | 0.701 | 0.765 |  |
| Left Middle Temporal | 0.110 | 0.385 | 0.986 | 0.061 | 0.630 | 0.844 | 0.125 | 0.326 | 0.765 |  |
| Abbrevations: SUMD, The Scale to Assess Unawareness in Mental Disorder; SZH, Patients with Schizophrenia | | | | | | | | | |  |

| **Supplementary Table 7.**  Correlations Between ICV Adjusted Volumes and SUMD in SZH (controlling for duration of illness, education, and medication) | | |
| --- | --- | --- |
| ICV-adjusted Volumes | **SUMD** | |
|  | *r* | *P_unadjusted_* |
| rh bankssts volume | 0.075 | 0.558 |
| rh caudal anterior cingulate volume | -0.092 | 0.470 |
| rh caudal middle frontal volume | -0.113 | 0.375 |
| rh cuneus volume | -0.031 | 0.806 |
| rh entorhinal volume | -0.112 | 0.378 |
| rh fusiform volume | 0.005 | 0.970 |
| rh inferior parietal volume | 0.151 | 0.233 |
| rh isthmus cingulate volume | 0.047 | 0.710 |
| rh lateral occipital volume | 0.058 | 0.652 |
| rh lateral orbitofrontal volume | -0.037 | 0.771 |
| rh lingual volume | -0.004 | 0.978 |
| rh medial orbitofrontal volume | -0.095 | 0.455 |
| rh parahippocampal volume | -0.087 | 0.493 |
| rh paracentral volume | -0.150 | 0.238 |
| rh pars opercularis volume | 0.055 | 0.668 |
| rh pars orbitalis volume | -0.062 | 0.625 |
| rh pars triangularis volume | 0.021 | 0.866 |
| rh pericalcarine volume | -0.124 | 0.330 |
| rh postcentral volume | 0.074 | 0.564 |
| rh posterior cingulate volume | 0.022 | 0.861 |
| rh precentral volume | -0.017 | 0.891 |
| rh praecuneus volume | 0.155 | 0.221 |
| rh rostral anterior cingulate volume | 0.046 | 0.718 |
| rh rostral middle frontal volume | -0.056 | 0.658 |
| rh superior frontal volume | 0.025 | 0.843 |
| rh superior parietal volume | 0.007 | 0.958 |
| rh supramarginal volume | 0.015 | 0.908 |
| rh frontal pole volume | 0.053 | 0.678 |
| rh temporal pole volume | 0.050 | 0.697 |
| rh transverse temporal volume | 0.154 | 0.224 |
| rh insula volume | -0.026 | 0.839 |
| lh bankssts volume | 0.005 | 0.968 |
| lh caudal anterior cingulate volume | 0.023 | 0.857 |
| lh caudal middle frontal volume | -0.065 | 0.612 |
| lh cuneus volume | 0.112 | 0.379 |
| lh entorhinal volume | -0.059 | 0.644 |
| lh fusiform volume | -0.039 | 0.758 |
| lh inferior temporal volume | -0.006 | 0.965 |
| lh isthmus cingulate volume | 0.083 | 0.513 |
| lh lateral occipital volume | 0.070 | 0.581 |
| lh lateral orbitofrontal volume | -0.071 | 0.579 |
| lh lingual volume | 0.050 | 0.696 |
| lh medial orbitofrontal volume | -0.081 | 0.526 |
| lh parahippocampal volume | -0.093 | 0.467 |
| lh paracentral volume | 0.017 | 0.894 |
| lh pars opercularis volume | 0.083 | 0.517 |
| lh pars orbitalis volume | -0.013 | 0.916 |
| lh pars triangularis volume | 0.007 | 0.958 |
| lh pericalcarine volume | 0.008 | 0.951 |
| lh postcentral volume | -0.034 | 0.790 |
| lh posterior cingulate volume | 0.115 | 0.365 |
| lh precentral volume | -0.098 | 0.443 |
| lh praecuneus volume | 0.150 | 0.238 |
| lh rostral anterior cingulate volume | -0.069 | 0.586 |
| lh rostral middle frontal volume | -0.089 | 0.484 |
| lh superior frontal volume | -0.104 | 0.414 |
| lh superior parietal volume | 0.136 | 0.282 |
| lh supramarginal volume | 0.058 | 0.648 |
| lh frontal pole volume | 0.108 | 0.395 |
| lh temporal pole volume | 0.072 | 0.573 |
| lh transverse temporal volume | 0.103 | 0.419 |
| lh insula volume | 0.052 | 0.686 |
| **FDR corrected p<0.05  *Abbreviations*. rh; right hemisphere; lh, left hemisphere; SZH, Schizophrenia; SUMD, The Scale to Assess Unawareness in Mental Disorder | | |
